# Antibiotic prescribing patterns, antimicrobial stewardship implementation barriers and readiness at primary health care centres in eastern Uttar Pradesh, India

**DOI:** 10.3389/fpubh.2026.1945074

**Published:** 2026-10-22

**Authors:** Neha Srivastava, Ashok Kumar Pandey, Laiba Jamal Ahmad, Umaer Alam, Rohit Beniwal, Niraj Kumar, Shailesh Kumar, Sakshi Singh, Hari Shanker Joshi

**Affiliations:** 1 Model Rural Health Research Unit (MRHRU Pali), ICMR-National Institute of Health Research, Gorakhpur, Uttar Pradesh, India; 2 Viral Immunology and Vaccine Research Division, ICMR- National Institute of Health Research, Gorakhpur, Uttar Pradesh, India; 3 Social and Behavioural Health Research Division, ICMR-National Institute of Health Research, Gorakhpur, Uttar Pradesh, India; 4 Molecular Biology Division, ICMR-National Institute of Health Research, Gorakhpur, Uttar Pradesh, India; 5 ICMR-National Institute of Health Research, Gorakhpur, Uttar Pradesh, India

**Keywords:** antibiotic prescribing, antimicrobial resistance, antimicrobial stewardship, primary healthcare, WHO AWaRe classification

## Abstract

**Background:**

Inappropriate antibiotic use in primary care is a major contributor to antimicrobial resistance, particularly in resource-constrained settings where empirical treatment is common. Community Health Centres (CHCs) provide first-referral care for much of rural India, yet evidence on antibiotic prescribing patterns and preparedness for antimicrobial stewardship in these settings is limited.

**Objective:**

We assessed paediatric outpatient antibiotic prescribing using the WHO Access, Watch, and Reserve (AWaRe) classification and evaluated clinicians’ knowledge, attitudes, and readiness to implement antimicrobial stewardship at rural CHCs in Gorakhpur, Uttar Pradesh, India.

**Methods:**

We conducted this cross-sectional study between February and December 2025. The quantitative component comprised a prescription audit of paediatric outpatients (≤18 years) presenting with acute febrile illness, acute respiratory infection, acute diarrhoeal disease or presumptive urinary tract infection at a rural CHC. Prescribed antibiotics were classified according to the WHO AWaRe framework. We also surveyed 40 clinicians from Community Health Centres across Gorakhpur district using the questionnaire which included closed-ended items assessing antimicrobial stewardship knowledge, attitudes and practices to identify perceived barriers to antimicrobial stewardship implementation. Quantitative data were summarized descriptively and used to inform the proposed antimicrobial stewardship implementation framework.

**Results:**

We reviewed 602 paediatric prescriptions. Antibiotics were prescribed in 98·8% of acute respiratory infections, 100% of acute diarrhoeal disease cases and 86·9% of acute febrile illness encounters. Only 18·8% of prescribed antibiotics belonged to the WHO Access category, substantially below the WHO target of at least 60%, whereas Watch antibiotics predominated across all clinical syndromes. Azithromycin was the most frequently prescribed antibiotic. Among clinicians, awareness of antimicrobial resistance was high (95%) and most were aware of national antimicrobial treatment guidelines (80%); however, familiarity with the WHO AWaRe classification (45%) and antimicrobial stewardship concepts (32·5%) was limited. Only six (33·3%) of the 18 clinicians familiar with AWaRe correctly identified Reserve antibiotics. Total 31 (77·5%) clinicians demonstrated a positive attitude towards antimicrobial stewardship and 92.5% clinicians considered implementation of a facility-level stewardship programme feasible despite reporting major barriers, including limited diagnostic capacity, absence of formal stewardship structures, workforce shortages and high patient workload.

**Conclusion:**

Paediatric outpatient antibiotic prescribing at a rural Community Health Centre in eastern Uttar Pradesh was characterised by extensive use of WHO Watch-category antibiotics and limited use of Access antibiotics. High awareness of antimicrobial resistance contrasted with important gaps in antimicrobial stewardship knowledge and stewardship infrastructure. These findings suggest that improving antibiotic use in scrub typhus–endemic primary-care settings will require context-specific antimicrobial stewardship programmes that combine clinician education, AWaRe-based prescribing indicators, strengthened diagnostic support and routine prescription audit and feedback.

## Introduction

World Health Organization (WHO) ranks antimicrobial resistance (AMR) among the top 10 public health threats (1). AMR is driven in part by the irrational and excessive use of antibiotics across healthcare systems (2). AMR has now emerged as one of the most pressing global public health threats. In low- and middle-income countries, antibiotic prescribing frequently occurs in settings with limited diagnostic infrastructure, high infectious disease burden and constrained stewardship oversight (3, 4).

India carries a substantial burden of infectious diseases and antimicrobial resistance (1, 4). Primary-care facilities represent a major source of antibiotic prescribing because they serve as the first point of contact for most acute infectious illnesses (4). Under the National Health Mission and Ayushman Bharat programme, Community Health Centres (CHCs) play a critical role in delivering primary and first-referral healthcare services in rural settings (4). However, these facilities often function under conditions of high patient load, limited laboratory support and reliance on empirical treatment. Consequently, strengthening antimicrobial stewardship at CHCs is essential to improve antibiotic use and preserve the effectiveness of existing antimicrobial agents.

In eastern Uttar Pradesh, acute febrile illnesses (AFI) progressing to acute encephalitis syndrome (AES) continue to be a major public health concern. The untreated or inadequately treated scrub typhus–positive AFI cases may progress to AES (5, 6). In response, the Government of Uttar Pradesh issued a guideline in 2016 recommending empirical treatment with azithromycin or doxycycline for suspected scrub typhus–associated febrile illness at CHCs, as delays in appropriate therapy can result in severe complications, particularly AES (6, 7). This epidemiological context strongly influences clinicians’ prescribing practices in the region. However, concerns persist regarding the widespread empirical use of broad-spectrum antibiotics for other outpatient conditions, including respiratory and diarrhoeal illnesses. A previous study by our team from this region reported substantial overprescription of WHO Watch category antibiotics at CHCs, largely driven by state treatment guidelines and clinicians’ concerns regarding progression to acute encephalitis syndrome (AES) (8).

To support rational antibiotic use and stewardship efforts, the WHO introduced the Access, Watch, and Reserve (AWaRe) classification framework, which prioritizes the use of narrow-spectrum Access antibiotics and recommends that at least 60% of national antibiotic consumption should derive from the Access category (9). Evidence from Indian studies at primary care facilities highlights that nearly 45–50% of outpatient antibiotic prescriptions in India reportedly involve WHO AWaRe “Watch” category antibiotics, despite recommendations favouring “Access” antibiotics for most common infections (4). The overprescription is driven by multiple interconnected factors such as limited access to point-of-care diagnostics, understaffed health facilities, weak regulatory oversight and widespread empirical prescribing practices. Evidence from standardized patient studies has shown that antibiotics are inappropriately prescribed in nearly half of clinical encounters involving conditions such as watery diarrhoea, presumed tuberculosis, and asthma, where antibiotics have no therapeutic benefit (10, 11). In rural settings, informal healthcare providers (IHCPs) further contribute to inappropriate use through entrenched prescribing beliefs, patient expectations for rapid recovery and easy over-the-counter availability of antibiotics (4, 12).

In addition to prescribing practices at rural CHC, successful antimicrobial stewardship implementation requires understanding of clinician knowledge, attitudes, operational barriers and readiness for stewardship activities within local healthcare contexts considering epidemiological complexities. Such information is particularly important for designing feasible stewardship interventions for resource-constrained primary-care settings. Therefore, this study aimed to characterize paediatric antibiotic prescribing patterns at a rural CHC using the WHO AWaRe classification, assess clinician knowledge, attitudes and readiness and perceived barriers toward antimicrobial stewardship implementation. We further used these findings to identify priorities for implementing a context-specific antimicrobial stewardship programme suitable for resource-constrained primary-care settings.

## Methods

### Study design

We conducted this cross-sectional study at a rural Community Health Centre (CHC) in Gorakhpur district, Uttar Pradesh, India, between February and December 2025. The study aimed to assess antibiotic prescribing patterns among paediatric outpatients, classify prescribed antibiotics according to the World Health Organization (WHO) Access, Watch, and Reserve (AWaRe) framework, and evaluate clinicians’ knowledge, attitudes, and readiness toward implementation of an antimicrobial stewardship programme (ASP) at the facility level.

The study consisted of two components: (1) a prescription audit of paediatric outpatient encounters and (2) a structured questionnaire survey of medical officers involved in outpatient antibiotic prescribing. Findings from both components were subsequently used to develop a context-specific antimicrobial stewardship framework tailored to the operational realities of the study facility.

This investigation was designed as a facility-level exploratory assessment and was not intended to generate population-representative estimates for all primary-care settings in India.

No formal sample size calculation was performed for the study. The sample size was based on the accessible eligible records within the defined study setting and study period rather than on a prospective hypothesis-testing sample size calculation.

### Study setting

The study was conducted at a Community Health Centre located in Gorakhpur district in eastern Uttar Pradesh, India. The facility provides outpatient primary and first-referral healthcare services, including maternal and child health services, to a predominantly rural population. The region is characterized by a high burden of infectious diseases and frequent empirical management of acute febrile illnesses in resource-constrained settings.

The study was implemented through the Model Rural Health Research Unit (MRHRU), Gorakhpur, which functions under the Department of Health Research (DHR), Ministry of Health and Family Welfare, Government of India (13). The MRHRU initiative was established to strengthen rural health research infrastructure, facilitate linkages between researchers and communities, and support translation of research evidence into rural healthcare practice.

### Study population and eligibility criteria

The quantitative component included paediatric outpatients aged ≤18 years presenting with one of four predefined infectious syndromes commonly associated with empirical antibiotic prescribing in outpatient settings:

- Acute respiratory infection (ARI)
- Acute diarrhoeal disease (ADD)
- Acute febrile illness (AFI)
- Presumptive urinary tract infection (UTI)

Patients presenting with conditions outside these predefined syndromic categories or with incomplete prescription records were excluded from analysis.

### Operational definitions

Standardized operational definitions were used to ensure consistency in identifying eligible cases.

Acute respiratory infection (ARI): acute onset illness (≤7 days duration) presenting with at least two of the following symptoms: fever, cough, sore throat, nasal congestion, or shortness of breath (14).

Acute diarrhoeal disease (ADD): passage of three or more loose or liquid stools per day, or more frequently than normal for the individual (15).

Acute febrile illness (AFI): fever lasting more than 3 days without an identifiable organ-specific cause at presentation (8).

Presumptive urinary tract infection (UTI): acute dysuria or fever accompanied by at least one of the following symptoms: urgency, frequency, suprapubic pain, gross haematuria, costovertebral angle tenderness, or urinary incontinence (16).

### Data collection

#### Prescription audit

Prescription audit data were collected from outpatient encounters between February and December 2025 using a structured data extraction form. Information extracted included patient age, presenting clinical syndrome, antibiotics prescribed, use of combination therapy and duration of antibiotic treatment. Only de-identified prescription data were recorded to maintain patient confidentiality.

#### Clinician’s survey

While prescription audit was done at single facility, we tried recruiting clinicians from all the 19 community health centres of Gorakhpur for survey using structured questionnaire. Our earlier study from Gorakhpur which included 16 CHCs provided the evidence of overprescription of antibiotics in this setting (8). Out of 19 CHCs, 11 CHC were purposively selected based on findings of earlier study (8), high footfall and geographical representativeness. Interviews were conducted with medical officers involved in outpatient clinical care and antibiotic prescribing at the 11 health facilities, i.e., Chargawan, Sahjanwa, Pali, Piprauli, Urwa, Khajni, Jungle Kaudiya, Chauri chaura, Pipraich, Bhathat, Harnahi. Participants were purposively selected based on their role in prescribing and patient management.

#### Questionnaire development

The questionnaire was developed using the National Treatment Guidelines for Antimicrobial Use in Infectious Disease Syndromes, WHO AWaRe guidance, principles of antimicrobial stewardship and published literature on ASP implementation in low- and middle-income settings (17–21).

The interview explored:

- Awareness and understanding of antimicrobial resistance (AMR)
- Familiarity with WHO AWaRe classification
- Knowledge of antimicrobial stewardship concepts
- Perceptions regarding antibiotic prescribing practices
- Barriers to rational antibiotic use
- Diagnostic and operational constraints
- Feasibility and acceptability of ASP implementation at the facility

#### Antibiotic classification

All prescribed antibiotics were categorized according to the WHO AWaRe classification framework (9).

- Access antibiotics: narrow-spectrum antibiotics recommended as first- or second-line treatment for common infections and intended to be widely available.
- Watch antibiotics: broader-spectrum antibiotics associated with higher resistance-selection potential and recommended only for specific indications.
- Reserve antibiotics: last-resort antibiotics intended for treatment of multidrug-resistant infections.

### Outcome measures

The primary outcomes were the distribution of Access, Watch and Reserve antibiotics among paediatric outpatient prescriptions and clinicians’ knowledge and attitudes regarding antimicrobial stewardship. Knowledge was assessed using six questions. Each correct response received one point, generating a total score ranging from 0 to 6. Scores were categorized as poor (0–2), moderate (3, 4), or good (5, 6). Attitudes toward antimicrobial stewardship were assessed using five point Likert-scale items strongly disagree to strongly agree for three items. Composite attitude scores ranged from 3 to 15 and were categorized as negative (3–8), neutral or moderately positive (9–11), and positive (12–15). Internal consistency of the attitude scale was assessed using Cronbach’s alpha.

### Data analysis

Quantitative data were analysed using descriptive statistics. Categorical variables were summarized using frequencies and percentages. The proportion of prescriptions containing Access, Watch, and Reserve antibiotics was calculated overall and by clinical syndrome category. Confidence intervals for selected prescribing proportions were estimated using binomial methods. Data analysis was performed using Stata MP version 2 Core (StataCorp, College Station, TX, USA).

Descriptive statistics was used to summarize participant characteristics, knowledge, attitudes, prescribing practices and antimicrobial stewardship (AMS) readiness. Categorical variables are presented as frequencies and percentages, while continuous variables are summarized using mean ± standard deviation or median (interquartile range), depending on data distribution.

Associations between clinician characteristics and stewardship readiness or prescribing practices was assessed using the Chi-square test or Fisher’s exact test for categorical variables and Student’s t-test or Mann–Whitney U test for continuous variables.

### Development of antimicrobial stewardship framework

Findings from the prescription audit and clinician’s interviews were integrated to develop a pragmatic antimicrobial stewardship framework tailored for implementation at the study facility. The framework was designed to address operational barriers identified during the study including limited diagnostic support, empirical prescribing practices, staff constraints and gaps in stewardship awareness. Rather than proposing a *de novo* stewardship model, the framework represents a context-specific adaptation designed for a rural Community Health Centre (CHC), focusing on strengthening rational antibiotic use within existing infrastructural and operational constraints. Feasibility ratings (‘High’ vs. ‘Moderate’) assigned to individual components reflect investigator assessment of operational complexity and resource availability: ‘High’ feasibility components leverage existing administrative workflows with minimal resource input, whereas ‘Moderate’ feasibility components require incremental training, staffing, or diagnostic infrastructure.

## Results

### Prescription audit and antibiotic prescribing patterns

A total of 602 outpatient prescriptions for children aged ≤18 years were reviewed between February and December 2025 (Table 1). Acute febrile illness (AFI) accounted for the largest proportion of encounters (336/602, 55.8%), followed by acute respiratory infections (ARI) (161/602, 26.7%), acute diarrhoeal disease (ADD) (71/602, 11.8%) and presumptive urinary tract infections (UTI) (34/602, 5.6%). Across all syndromes, antibiotic prescribing was highly prevalent and empirical treatment with WHO Watch-category antibiotics predominated.

**Table 1:** Characteristics of outpatient encounters included in prescription audit.

Variable	*n* (%)
Total prescriptions reviewed	602
Acute febrile illness (AFI)	336 (55.8)
Acute respiratory infection (ARI)	161 (26.7)
Acute diarrheal disease (ADD)	71 (11.8)
Presumptive urinary tract infection (UTI)	34 (5.6)
Antibiotic prescribed	546 (90.7)
No antibiotic prescribed	56 (9.3)

Antibiotics were prescribed in 546 of 602 encounters (90.7%, 95% CI 88.3–92.9). Prescribing frequency varied by syndrome, ranging from 70.6% in presumptive UTI cases to 100% in acute diarrhoeal disease cases (Table 2). Among children presenting with AFI (*n* = 336), antibiotics were prescribed in 292 cases (86.9%). Azithromycin was the most frequently prescribed antibiotic (42.6%), followed by cefixime (14.9%). Combination antibiotic therapy was observed in 16.0% of AFI encounters, most commonly azithromycin combined with cefixime or amoxicillin. Access-category antibiotics accounted for only a small proportion of prescriptions, primarily amoxicillin (8.6%) and doxycycline (3.3%). In ARI cases (*n* = 161), antibiotics were prescribed in 159 encounters (98.8%). Azithromycin (47.2%) was the most prescribed antibiotic, followed by amoxicillin (26.1%). Combination therapy was prescribed in 16.1% of ARI cases, predominantly involving azithromycin-based regimens.

**Table 2:** Antibiotic prescribing patterns across clinical syndromes by AWaRe classification.

Antibiotic regimen	AWaRe	AFI (*n* = 336) *n* (%)	ARI (*n* = 161) *n* (%)	ADD (*n* = 71) *n* (%)	UTI (*n* = 34) *n* (%)
No antibiotic	—	44 (13.1)	2 (1.2)	0 (0.0)	10 (29.4)
Single antibiotic					
Azithromycin	Watch	143 (42.6)	76 (47.2)	16 (22.5)	10 (29.4)
Doxycycline	Access	11 (3.3)	2 (1.2)	2 (2.8)	0 (0.0)
Amoxicillin	Access	29 (8.6)	42 (26.1)	0 (0.0)	0 (0.0)
Cefixime	Watch	50 (14.9)	11 (6.8)	5 (7.0)	5 (14.7)
Levofloxacin	Watch	7 (2.1)	2 (1.2)	0 (0.0)	0 (0.0)
Amikacin	Watch	1 (0.3)	0 (0.0)	0 (0.0)	0 (0.0)
Ciprofloxacin	Watch	4 (1.2)	0 (0.0)	0 (0.0)	0 (0.0)
Ofloxacin	Watch	1 (0.3)	0 (0.0)	2 (2.8)	3 (8.8)
Metronidazole	Access	0 (0.0)	0 (0.0)	27 (38.0)	0 (0.0)
Norfloxacin	Watch	0 (0.0)	0 (0.0)	11 (15.5)	0 (0.0)
Combination antibiotics					
Azithro + Doxy	Watch + Access	7 (2.1)	11 (6.8)	2 (2.8)	3 (8.8)
Azithro + Cefixime	Watch + Watch	18 (5.4)	9 (5.6)	5 (7.0)	0 (0.0)
Azithro + Amoxicillin	Watch + Access	11 (3.3)	6 (3.7)	0 (0.0)	0 (0.0)
Azithro + Levofloxacin	Watch + Watch	3 (0.9)	0 (0.0)	0 (0.0)	0 (0.0)
Azithro + Ofloxacin	Watch + Watch	3 (0.9)	0 (0.0)	0 (0.0)	0 (0.0)
Doxy + Cefixime	Access + Watch	1 (0.3)	0 (0.0)	0 (0.0)	0 (0.0)
Amoxicillin + Amikacin	Access + Watch	3 (0.9)	0 (0.0)	0 (0.0)	0 (0.0)
Azithro + Ceftriaxone	Watch + Watch	0 (0.0)	0 (0.0)	1 (1.4)	3 (8.8)

Values are presented as number (percentage). Percentages are calculated column-wise within each clinical syndrome. AFI, Acute febrile illness; ARI, Acute respiratory infection; ADD, Acute diarrheal disease; UTI, Urinary tract infection. AWaRe classification as per WHO 2023.

Watch-category antibiotics were prescribed singly or in combination in 433 (79.3%; 95% CI 75.6–82.6) out of 546 antibiotics prescribed across all syndromes. Among children with ADD (*n* = 71), antibiotics were prescribed in all encounters. Metronidazole (38.0%) was the most frequently prescribed antibiotic, followed by azithromycin (22.5%) and norfloxacin (15.5%). Combination antibiotic therapy was observed in 11.3% of cases. Watch-category antibiotics were commonly prescribed despite routine antibiotic therapy not being recommended for many acute diarrhoeal illnesses. In presumptive UTI cases (*n* = 34), antibiotics were prescribed in 24 encounters (70.6%). Azithromycin was the most prescribed antibiotic (29.4%), followed by cefixime (14.7%) and ofloxacin (8.8%). Combination therapy was prescribed in 17.6% of UTI cases.

Watch-category antibiotics dominated prescribing patterns across all clinical syndromes (Table 2).

### WHO AWaRe classification of prescribed antibiotics

Analysis using the WHO AWaRe framework demonstrated substantial predominance of Watch-category antibiotics in outpatient prescribing. Only 18.8% of prescribed antibiotics belonged to the Access category, whereas most prescriptions involved Watch-category agents. Macrolides represented the most frequently prescribed antibiotic class, accounting for 56.5% of all antibiotic prescriptions, largely driven by extensive azithromycin use (Table 3). Cephalosporins constituted the second most common antibiotic class (18.6%), followed by penicillins (15.1%). Fluoroquinolones accounted for 5.5% of prescriptions. Reserve antibiotics were not identified in routine prescribing during the study period.

**Table 3:** Overall antibiotic utilization by antibiotic class and WHO AWaRe category.

Antibiotic class	Common antibiotics included	AWaRe category	Frequency (n)	Percentage (%)
Macrolides	Azithromycin (alone and in combinations)	Watch	340	56.5
Cephalosporins (oral and parenteral)	Cefixime, Ceftriaxone	Watch	112	18.6
Penicillin	Amoxicillin	Access	91	15.1
Nitroimidazoles	Metronidazole	Access	27	4.5
Fluoroquinolones	Ciprofloxacin, Levofloxacin, Ofloxacin, Norfloxacin	Watch	33	5.5
Tetracyclines	Doxycycline	Access	18	3.0
Aminoglycosides	Amikacin	Watch	4	0.7
No antibiotic prescribed	—	—	56	9.3

The denominator was 602 encounters. As some encounters involved combination antibiotic therapy, more than one antibiotic could be prescribed per encounter. Therefore, the frequencies and percentages of antibiotic classes are not mutually exclusive and may exceed the total number of encounters or 100%, respectively.

#### Clinician characteristics and antimicrobial stewardship knowledge

Forty healthcare providers participated in the study, including 28 general physicians, 6 gynecologist, 1 pediatrician, 2 ophthalmologist and 3 dental surgeons. Clinical experience ranged from 3 months to 12 years, and reported outpatient patient load ranged from 10 to 100 patients per clinician per day. Total 38 out of 40 clinicians were aware of antimicrobial resistance (AMR). About 80% participants reported familiarity with national treatment guideline and only 45% had awareness of the WHO AWaRe classification system. About 72.5% clinicians were aware of regional scrub typhus treatment recommendations issued by the Government of Uttar Pradesh. However, understanding of formal antimicrobial stewardship programme (ASP) concepts was only in 32.5%. Only 33.3% of clinicians reported having knowledge of AWaRe classification were able to identify Reserve-category antibiotic correctly (Table 4).

**Table 4:** Knowledge and awareness of antimicrobial stewardship among healthcare providers (*n* = 40).

Variable	Yes *n* (%)
Aware of antimicrobial resistance (AMR)	38 (95)
Familiar with WHO AWaRe classification (Access, Watch, and Reserve) of antibiotics	18 (45)
Aware of national treatment guidelines for antimicrobial use in infectious disease syndromes (2025)	32 (80)
Aware of government of Uttar Pradesh empirical antibiotic guideline for suspected scrub typhus (2016)	29 (72.5)
Familiar with antibiotic stewardship protocol (ASP) concept	13 (32.5)
Able to identify reserve category antibiotics correctly (*n* = 18)	6 (33.3)

The median knowledge score was 3 (IQR 3–4) out of a maximum possible score of 6. Overall, 9 (22.5%) clinicians had good knowledge, 16 (40%) had moderate knowledge, and 15 (37.5%) had poor knowledge.

Table 5 shows the association between clinicians’ clinical characteristics and their level of knowledge. No distinct descriptive patterns emerged between daily patient load and knowledge categories.

**Table 5:** Association between clinicians’ characteristics and knowledge level among clinicians.

Clinicians characteristics	Good knowledge (*N* = 9)	Moderate knowledge (*N* = 16)	Poor knowledge (*N* = 15)
Designation of clinician			
General physician	7 (77.7%)	11 (68.7%)	10 (66.7%)
Gynecologist	0	3 (18.7%)	3 (20%)
Dental Surgeon	0	1 (6.25%)	2 (13.3%)
Ophthalmologist	1 (11.1%)	1 (6.25%)	0
Pediatrician	1 (11.1%)	0	0
Years of clinical experience			
Less than a year	2 (22.2%)	6 (37.5%)	4 (26.7%)
2–5 years	5 (55.5%)	8 (50%)	8 (53.3%)
>5 years	2 (22.2%)	2 (12.5%)	3 (20%)
Mode of working			
Full time	8 (88.9%)	16 (100%)	13 (86.7%)
Part time	1 (11.1%)	0	2 (13.3%)
Daily patient load			
10–20	1 (11.1%)	3(18.7%)	3 (20%)
21–30	1(11.1%)	2(12.5%)	3 (20%)
31–40	2(22.2%)	3(18.7%)	3 (20%)
41–50	1(11.1%)	5 (31.2%)	2 (13.3%)
51–60	0	0	0
61–70	2(22.2%)	1(6.25%)	1 (6.7%)
71–100	2(22.2%)	2(12.5%)	3 (20%)

*p* values were obtained using the Chi-square test. If any expected cell count was less than 5, Fisher’s exact test was used.

#### Attitudes toward antimicrobial stewardship and prescribing practices

The internal consistency of the 3-item attitude scale was evaluated using Cronbach’s alpha, yielding α=0.750 indicating acceptable internal reliability. Overall attitudes toward antimicrobial stewardship were positive. Total 34 (85%) participants agreed that formal ASP implementation was needed at the facility level and all 40 (100%) clinicians believed that adherence to treatment guidelines could improve patient outcomes. Total 34 (85%) participants also expressed interest in receiving structured training on rational antibiotic prescribing and stewardship practices. Despite confidence in clinical prescribing decisions by 31(77.5%) clinicians, they acknowledged operational limitations affecting prescribing behaviour including diagnostic uncertainty and high patient burden.

The median attitude score was 12 (IQR 12–13). Total 31 (77.5%) clinicians demonstrated a positive attitude toward antimicrobial stewardship, 8 (20%) had a neutral/moderately positive attitude and 1 (2.5%) had a negative attitude.

#### Barriers to antimicrobial stewardship implementation

Antibiotic prescribing in outpatient settings was common, with reported frequency ranging from sometimes (10–30% of patients) to very often (>60%). Choice of antibiotics was primarily influenced by clinician habit or experience (30%), national or state guidelines (27.5%), lab culture results (12.5%), drug availability (12.5%) and facility level policy (2.5%). All participants reported prescribing antibiotics empirically, with empirical prescribing occurring sometimes (47.5%) to frequently (12.5%) depending on the clinical syndrome. Documentation of the indication and duration of antibiotic therapy was recorded by 57.5% clinicians and routine review of antibiotic prescriptions after 48–72 h was reported by 77.5% clinicians.

Participants reported the absence of formal antimicrobial stewardship structures at the facility level. No dedicated ASP focal person, formal stewardship committee or routine antibiotic audit mechanism was Available online at the time of the study.

Several barriers to ASP implementation were identified (Table 6):

- Lack of scrub typhus serological testing at facilities by 87.5% clinicians
- Lack of basic microbiology lab by 80% clinicians
- Non availability of quick lab culture and sensitivity report by 85% clinicians
- Shortage of trained personnel
- High outpatient patient load
- Inconsistent antibiotic supply systems
- Inadequate documentation and monitoring systems
- Patient expectations for antibiotic treatment

**Table 6:** Barriers and facilitators to ASP implementation at CHC level.

Domain	Findings
Diagnostic barriers	Lack of microbiology support, no culture testing, lack of scrub typhus serological testing
Workforce barriers	Staff shortages, high patient load
System barriers	No audit systems, no ASP focal person
Behavioural barriers	Patient expectation for antibiotics
Facilitators	Positive attitude toward ASP, willingness for training, ready to attend and participate in ASP at their facility, urgent need of ASP reported by clinicians

Despite these constraints, 92.5% participants considered implementation of a facility-level antimicrobial stewardship programme feasible if supported through institutional leadership, training and operational guidance.

All participants expressed willingness to participate in antibiotic stewardship training and chose e-learning or online sessions (65%) as most suitable mode followed by on-site training (35%). Participants identified senior clinicians (60%) as potential facilitators for stewardship training activities followed by microbiologists (42.5%) and district-level trainers (40%).

#### AWaRe classification of antibiotics in list of essential medicines for community health Centre (CHC)

Of the 41 antibiotics listed in the Essential Medicines List (EML) for Community Health Centres (22), 18 (43.9%) were classified as Access antibiotics, 15 (36.6%) as Watch, and 8 (19.5%) as Reserve according to the WHO AWaRe classification. Although Access antibiotics are available within the public health system, our findings demonstrated predominant use of Watch-category antibiotics, suggesting that prescribing of watch category antibiotics is unlikely to be explained solely by medicine availability.

#### Development of a context-specific antimicrobial stewardship framework

Findings from the prescription audit and clinicians’ interviews were integrated and interpreted alongside established antimicrobial stewardship principles in India 18,34, and WHO guidance for antimicrobial stewardship implementation in resource-constrained settings to develop a pragmatic, facility-level framework tailored to the study setting (Table 7). Rather than proposing a *de novo* stewardship model, the framework represents a context-specific adaptation designed for a rural Community Health Centre (CHC), focusing on strengthening rational antibiotic use within existing infrastructural and operational constraints. The proposed framework prioritizes simplified syndromic treatment algorithms, promotion of Access-category antibiotics where clinically appropriate, clinician training, prescription audit and feedback mechanisms and incremental strengthening of diagnostic support.

**Table 7:** Context-adapted CHC-level antimicrobial stewardship implementation framework based on study findings and existing stewardship principles.

Component	Proposed intervention	Feasibility	Why?
Leadership	ASP focal person	Moderate	Requires identifying and training a dedicated staff member and ensuring leadership support which may not always be Available online at CHCs.
Prescribing policy	Access-first antibiotic policy	High	Can be implemented through local prescribing guidelines with minimal additional resources or infrastructure.
Monitoring	Monthly audit and feedback	Moderate	Requires regular collection, analysis, and review of prescribing data, demanding staff time and organizational commitment.
Capacity building	Short stewardship training sessions	High	Short training sessions can be integrated into routine staff meetings and require relatively few resources.
Decision support	Syndromic treatment algorithms	High	Easy to implement by displaying or distributing standardized treatment algorithms, with minimal cost.
Surveillance	Prescription monitoring	Moderate	Requires systematic recording and analysis of antibiotic prescriptions, which may be limited by staffing, record-keeping systems or digital infrastructure.

Framework components are informed by study findings and aligned with established antimicrobial stewardship domains, including leadership, prescribing guidance, monitoring, education, and surveillance.

## Discussion

This study provides a facility-level assessment of antibiotic prescribing practices, WHO AWaRe antibiotic utilization and readiness for antimicrobial stewardship programme (ASP) implementation at a primary-care facility in eastern Uttar Pradesh, India. The findings demonstrate substantial reliance on Watch-category antibiotics, limited use of Access antibiotics, frequent empirical prescribing and absence of structured stewardship systems, while simultaneously identifying high clinician awareness of antimicrobial resistance (AMR) and positive acceptability toward ASP implementation.

A major finding of this study was the predominance of Watch-category antibiotics in outpatient prescribing. Only 18.8% of all prescribed antibiotics belonged to the WHO AWaRe Access group., While the WHO-recommended target that at least 60% of antibiotic consumption should derive from Access antibiotics (9, 23) which was formulated as a national-level metric, applying this threshold as a local institutional quality benchmark underscores a severe overreliance on Watch-category drugs in routine outpatient care.

Azithromycin and cephalosporins constituted many prescriptions across all syndromes, particularly among acute febrile illness (AFI) and acute respiratory infection (ARI) cases. Such prescribing patterns are concerning because Watch antibiotics possess greater resistance-selection potential and are intended for more restricted use under WHO stewardship guidance (9). The predominance of Watch-category antibiotics observed in this study is consistent with emerging evidence from India demonstrating high outpatient use of broad-spectrum antibiotics at the primary-care level (4, 8, 20). A recent systematic review of antibiotic prescribing practices in Indian primary-care settings reported widespread overuse of broad-spectrum agents and poor adherence to rational prescribing recommendations, particularly for respiratory and febrile illnesses (4). Similar prescribing trends have also been reported from low- and middle-income countries where diagnostic uncertainty, high patient burden and limited stewardship infrastructure have been reported alongside empirical broad-spectrum antibiotic use (24–27).

However, interpretation of prescribing patterns in this setting requires careful consideration of the local epidemiological context. Eastern Uttar Pradesh experiences a substantial burden of acute undifferentiated febrile illnesses due to scrub typhus mainly during monsoon and post-monsoon season (5). In such settings, empirical azithromycin or doxycycline therapy is commonly recommended in regional and state-level treatment guidance because delayed treatment of scrub typhus may lead to severe complications (AES) and mortality (6–8). Therefore, at least part of the observed empirical prescribing among AFI cases likely reflects attempts by clinicians to manage potentially serious endemic infections in the context of limited diagnostic support.

This contextual reality has important implications for antimicrobial stewardship implementation in resource-limited endemic settings. Stewardship interventions in such settings should not focus solely on reducing antibiotic prescription but rather on optimizing empirical antibiotic use through syndrome-based risk stratification, documentation, review mechanisms and context-adapted implementation of WHO AWaRe principles. In facilities where microbiological confirmation is largely unavailable, rigid restriction-based stewardship models may not be operationally feasible and could inadvertently compromise timely management of severe febrile illnesses. Instead, stewardship approaches should prioritize rational empirical prescribing, encourage Access-first therapy whenever clinically appropriate and strengthen systems for antibiotic review and de-escalation.

An important stewardship concern identified in the present study was the extensive use of azithromycin across multiple syndromes, including ARI, diarrhoeal disease, and presumptive urinary tract infections. A critical distinction must be made regarding the clinical context of its use like in endemic regions, empirical azithromycin or doxycycline for AFI compatible with scrub typhus (*Orientia tsutsugamushi*) is clinically justified and supported by national/state guidelines. In contrast, its widespread empirical administration for ARI, Acute Diarrheal Disease, and presumptive Urinary Tract Infections lacks guideline support. Most ARI and ADD cases are viral or self-limiting, while typical Gram-negative uropathogens causing UTIs do not achieve therapeutic susceptibility from macrolides due to poor urinary concentrations. While azithromycin may be justified for selected AFI cases in endemic regions, its widespread empirical use across syndromes where bacterial infection is uncertain may contribute to unnecessary selective pressure for macrolide resistance (28, 29). Furthermore, routine antibiotic prescribing for acute diarrhoeal disease and near-universal prescribing for respiratory infections suggest that opportunities exist to reduce unnecessary antibiotic exposure through syndrome-specific stewardship interventions and improved adherence to treatment guidelines. The study also identified important operational and structural barriers that may be associated with current prescribing behaviour. Clinicians consistently reported lack of microbiological facilities, absence of culture and sensitivity testing, limited availability of rapid diagnostics, high patient load and shortages of trained personnel. Similar barriers have been widely reported in primary-care and rural healthcare settings across low- and middle-income countries, where empirical antibiotic prescribing has been reported in association withconstrained diagnostic capacity and heavy outpatient workloads (30, 31). The absence of formal stewardship infrastructure, facility-level antibiotic policies and prescription audit systems further limits opportunities for systematic monitoring and rationalization of antibiotic use.

Despite these constraints, clinicians demonstrated high awareness of AMR and generally positive attitudes toward stewardship interventions. Most participants expressed willingness to participate in audit and feedback activities, guideline-based prescribing initiatives and structured stewardship training. These findings are important because provider engagement and acceptability are recognized as major facilitators of successful ASP implementation (32). However, knowledge of formal ASP concepts and recognition of Reserve antibiotics remained low, suggesting that stewardship education at the primary-care level should extend beyond general AMR awareness to include practical operational understanding of AWaRe classification, stewardship principles and syndrome-based prescribing approaches. These findings align with previous studies showing that clinicians may recognize AMR as a public health problem while lacking formal training in stewardship implementation frameworks (33–35).

The AWaRe distribution of antibiotics within the Community Health Centre Essential Medicines List (EML) also provides important insight into prescribing behaviour observed in this study. Less than half of the listed antibiotics belonged to the Access category, while Watch and Reserve antibiotics together constituted more than half of formulary antibiotics. This formulary composition may unintentionally facilitate broader use of Watch-category agents at primary-care level. The availability of Reserve antibiotics at CHC level, despite limited stewardship oversight and lack of microbiological support, further raises concerns regarding potential inappropriate use. Aligning CHC formularies more closely with WHO AWaRe recommendations and promoting Access-first availability may therefore represent an important systems-level stewardship intervention.

The findings of this study support development of a contextualized antimicrobial stewardship framework tailored for primary-care facilities managing endemic acute febrile illnesses in resource-constrained settings. Such a framework should integrate local epidemiology, syndromic risk stratification, AWaRe-informed prescribing, simplified treatment algorithms and pragmatic monitoring mechanisms. Rather than attempting to eliminate empirical therapy entirely, stewardship efforts should focus on improving appropriateness of empirical prescribing by strengthening documentation of indications, encouraging scheduled antibiotic review, limiting unnecessary combination therapy and promoting use of Access antibiotics where clinically feasible. Seasonal stewardship approaches adapted to periods of increased scrub typhus transmission may also be particularly relevant in endemic regions.

At the implementation level, feasible interventions may include designation of a facility-level ASP focal person, introduction of simplified syndromic treatment pathways, regular prescription audit with feedback, periodic AWaRe monitoring, and targeted clinician training. Even in the absence of advanced microbiology laboratories, basic stewardship indicators such as proportion of Access antibiotic use, frequency of azithromycin prescribing, and documentation of antibiotic indication could provide practical monitoring metrics for primary-care facilities.

This study has several strengths. It provides real-world prescribing data from a rural primary-care setting, incorporates WHO AWaRe classification for standardized stewardship assessment, and combines prescribing audit findings with clinician’s assessment of provider readiness and implementation barriers. Additionally, the study moves beyond descriptive antibiotic utilization by proposing a context-sensitive operational framework for ASP implementation in endemic AFI settings.

However, several limitations should be acknowledged. The study was conducted at a single facility for study prescription pattern and involved a relatively small number of clinicians limited to Gorakhpur district, limiting generalizability to other primary-care settings of other parts of India. Prescription appropriateness could not be definitively assessed because microbiological confirmation and patient-level outcome data were unavailable. The study focused primarily on outpatient paediatric syndromes and may not reflect prescribing practices in adult or inpatient populations. Because the study was exploratory and implementation-focused, it was not designed to establish causal relationships between stewardship determinants and prescribing behaviour. Additionally, the questionnaire used in the clinician survey was developed as a multidomain, study-specific assessment instrument rather than as a single standardized psychometric scale. Formal quantitative content-validity assessment was not performed. Similarly, formal construct-validity testing using exploratory or confirmatory factor analysis was not undertaken. Face-validity assessment and formal pilot testing were also not conducted as separate methodological procedures before the main survey. Internal consistency was assessed for the attitude domain using Cronbach’s alpha. Because the remaining questionnaire sections primarily consisted of factual knowledge questions, individual prescribing-practice questions, facility/resource questions and implementation-preference questions, they were not combined into a single scale for internal-consistency analysis.

The purposive selection of 11 of the 19 CHCs may have introduced facility-level selection bias, and therefore the findings on clinicians’ antimicrobial stewardship knowledge may not be fully generalizable to clinicians working in all CHCs across Gorakhpur district.

## Conclusion

This study identified substantial reliance on WHO Watch-category antibiotics and widespread empirical prescribing in paediatric outpatient care at a rural Community Health Centre in eastern Uttar Pradesh. Prescribing practices appeared to be strongly influenced by local epidemiological realities, limited diagnostic infrastructure, and operational constraints within the primary-care setting. Despite these challenges, clinicians demonstrated positive attitudes toward antimicrobial stewardship activities and expressed willingness to engage in stewardship interventions, suggesting important opportunities for implementation of context-appropriate stewardship strategies. The study further developed a pragmatic antimicrobial stewardship framework tailored to the operational realities of the study facility, emphasizing feasible interventions such as Access-first prescribing, syndrome-based treatment guidance, prescription audit with feedback, targeted clinician training, and strengthened diagnostic referral support. These findings support the need for locally adapted antimicrobial stewardship approaches that are feasible within resource-constrained rural primary-care environments. The proposed antimicrobial stewardship implementation framework should be considered a conceptual framework at this stage, as it has not yet been formally validated or evaluated for implementation effectiveness, prospective implementation research is needed to assess its feasibility, acceptability, sustainability, and impact on antibiotic prescribing practices. Future multicentre studies evaluating implementation effectiveness, prescribing appropriateness, and clinical outcomes are needed to strengthen evidence for scalable stewardship interventions in similar settings.

## Data Availability

The original contributions presented in the study are included in the article/supplementary material, further inquiries can be directed to the corresponding author/s.
